# Antiradical and Cytoprotective Activities of Several *C*-Geranyl-substituted Flavanones from *Paulownia tomentosa* Fruit

**DOI:** 10.3390/molecules15096035

**Published:** 2010-08-31

**Authors:** Aleš Zima, Jan Hošek, Jakub Treml, Jan Muselík, Pavel Suchý, Gabriela Pražanová, Ana Lopes, Milan Žemlička

**Affiliations:** 1 Department of Natural Drugs, Faculty of Pharmacy, University of Veterinary and Pharmaceutical Sciences Brno, Brno, Czech Republic; 2 Department of Pharmaceutics, Faculty of Pharmacy, University of Veterinary and Pharmaceutical Sciences Brno, Brno, Czech Republic; 3 Department of Human Pharmacology and Toxicology, Faculty of Pharmacy, University of Veterinary and Pharmaceutical Sciences Brno, Brno, Czech Republic; 4 Faculty of Pharmacy, University of Lisbon, Lisbon, Portugal

**Keywords:** *Paulownia tomentosa*, prenyl, flavanone, antiradical, cytoprotective

## Abstract

Antiradical and cytoprotective activities of several flavanones isolated from *Paulownia tomentosa* (Thunb.) Steud. (Scrophulariaceae) have been evaluated using different *in vitro* and *in vivo* methods. The capacity of flavanones to scavenge radicals was measured *in vitro* by means of DPPH and ABTS assays, the inhibition of hydroxyl radicals produced in Fenton reactions, FRAP, scavenging superoxide radicals using enzymatic and nonenzymatic assays and the inhibition of peroxynitrite-induced nitration of tyrosine. The *in vivo* testing involved measuring the cytoprotective effect of chosen flavanones against alloxan-induced diabetes in mice. The activity of tested compounds was expressed either as a Trolox^®^ equivalent or was compared with rutin or morine as known antioxidant compounds. The highest activity in most tests was observed for diplacone and 3´-*O*-methyl-5´-hydroxydiplacone, and the structure vs. the antioxidant activity relationship of geranyl or prenyl-substituted flavonoids with different substitutions at the B and C ring was discussed.

## 1. Introduction

The so-called civilization diseases are, especially in the Western world, complex illnesses resulting from bad and unhealthy life styled as well as impaired environmental conditions. The incidence of civilization disease is also connected to the increase of the World’s population and extended life-times. Such diseases are, for example, neurodegenerative disorders (senile dementia, Alzheimer’s disease), cardiovascular illnesses, diabetes mellitus type 2, and cancer. It is now common knowledge that the formation of reactive oxygen species (ROS) and/or nitric species (RNS) is highly implicated in the pathogenesis of such diseases [1,2]. ROS/RNS can be both harmful and beneficial in humans. The beneficial effects of ROS involve, for instance, the defense against infectious agents and several cellular signaling systems. In contrast, when released in high concentration, ROS/RNS can inflict damage to cellular components (lipids, membranes, nucleic acids, etc.). This situation can lead to oxidative stress. Oxidative stress has been defined as “a disturbance in the pro-oxidant and antioxidant balance in favor of the former, leading to potential damage” [2]. To counterbalance the harmful effects of ROS/RNS, organisms are protected by different antioxidants systems, which inhibit or retard the oxidation damage of cells and/or physiological processes [3,4].

Flavonoids are a structurally variable group of polyphenolic compounds that are ubiquitous in Nature. Up until the present, over 8,000 flavonoids have been identified [5]. The beneficial health effects of flavonoids are especially attributed to their antioxidant activity. Attempts to establish the relationship between structure and their radical-scavenging capacity have been successful [5,6,7]. The activity of flavonoids depends on the position of hydroxy, methoxy, geranyl or other group substitutions. The ability to act as an efficient antioxidant does not depend solely on the availability of phenolic hydrogens but also on the possible stabilization of the resulting radical [7,8].

*Paulownia tomentosa* (Thunb.) Steud. (Srophulariaceae) is a potent source of biologically active *C*-geranyl flavanones. According to scientific literature, these compounds have shown cytotoxic, antibacterial and antiradical properties [9,10,11]. In this present work, flavonoids with a geranyl and prenyl substitution isolated from *P. tomentosa* were evaluated *in vitro* in antiradical assays (DPPH, ABTS, the inhibition of Fenton reaction, inhibition of peroxynitrite induced nitration of tyrosine, FRAP, and the inhibition of superoxide) and *in vivo* for their cytoprotective effects against alloxan-induced diabetes.

## 2. Results and Discussion

The ability of variously substituted flavonoids from *P. tomentosa,* with a geranyl or prenyl chain at position 6, to act as potential antioxidants was tested. The tested flavanones showed different degrees of activity in all of the assays that were used. The activity of these compounds was expressed as TEAC (Trolox Equivalent Antioxidant Capacity) for ABTS, DPPH, FRAP, and the inhibition of peroxynitrite induced tyrosine nitration. The TEAC value is based on the ability of an antioxidant to scavenge radicals relative to the radical scavenging ability of the water-soluble vitamin E analogue, Trolox^®^ [12], and is expressed as multiples of the activity of Trolox^®^. Rutin was used as the standard of activity for comparing the effects in assays based on the inhibition of superoxide radical and inhibition of Fenton reaction. Morine was used as a reference compound in the alloxan-induced pancreatic damage test.

The evaluated compounds all had a flavanone skeleton except **5** and **10** (flavanonol structures). Compounds **1**-**9** were substituted with a geranyl side chain at the position 6 (hydroxylated geranyl present at **4** and **8**), **10** was substituted by a prenyl chain at the same position. The compounds had different substitution at the B and C ring (Table 1).

**Table 1:** Structures of compounds tested.

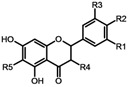					
Compound / substituent	R1	R2	R3	R4	R5
**Diplacone (1)**	OH	OH	H	H	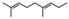
**Mimulone (2)**	H	OH	H	H	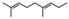
**3´-*O*-methyldiplacone (3)**	OMe	OH	H	H	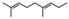
**Tomentodiplacone (4)**	OMe	OH	H	H	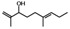
**3´-*O*-methyldiplacol (5)**	OMe	OH	H	OH	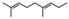
**3´-*O*-methyl-5´-OH-diplacone (6)**	OMe	OH	OH	H	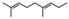
**3´-*O*-methyl-5´-*O*-methyldiplacone (7)**	OMe	OH	OMe	H	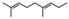
**Tomentodiplacone B (8)**	OMe	OH	H	H	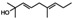
**Schizolaenone C (9)**	OH	H	OH	H	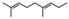
**6-isopentenyl-3´-*O*-methyltaxifolin (10)**	OMe	OH	H	OH	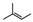

### 2.1. ABTS, DPPH, FRAP, the inhibition of peroxynitrite induced tyrosine nitration

Both the ABTS and DPPH tests identified the promising activity of compound **1** (TEAC_ABTS_ 3.2 and TEAC_DPPH_ 1.06) and compound **6** (TEAC_ABTS_ 1.66 and TEAC_DPPH_ 0.98). The reducing potential of the compounds (**4**, **8** and **9** were excluded because of insufficient material), was evaluated in the FRAP assay, where compounds **7** (1.189) and **6 **(0.741) showed the highest activity. The ability of the tested compounds to decrease peroxynitrite-induced nitration of tyrosine was investigated as well. The tested compounds showed similar results of activity. The highest ability to prevent against tyrosine nitration showed **9**. The results of the above-mentioned experiments are summarized in Table 2.

The structure and antioxidant activity relationships of flavonoids is determined by a) the *ortho* 3´,4´-dihydroxy substitution in the B ring, b) the *meta* 5,7-dihydroxy substitution in the A ring, c) the 2,3-double bond in C ring, d) the 4-keto substitution in C ring, e) the 3-hydroxyl group in the C ring. The spatial arrangement of the substitution is perhaps a greater determinant of antioxidant activity than the flavan skeleton [13,14]. Another substitution influencing antioxidant activity is *O*-methylation, which may decrease antioxidant activity [15]. Steric effects may also induce the suppression of antioxidant activity; the B ring is particularly sensitive to the position of the methoxy group and 2´-*O*-methyl, 4´-hydroxy substitution at the flavonoid B ring abolishes antioxidant activity, whereas 2´-hydroxy, 4´-*O*-methyl derivatives show activity. The methylation of the 3´,4´ *ortho* dihydroxy group leads to the decrease of scavenging [16]. Similar structure–activity relationships can also be deduced from the results presented here. Compounds with an *ortho*-dihydroxy substitution of the flavanone B-ring showed the highest activity, but the activity of methoxylated compounds (**3**, **4**, **5**, **7**, **8**) or compound with *para* located hydroxyl (**2**) was much lower. The 3-OH substitution of **5** did not affect activity, similar to the hydroxy substitution of the geranyl side chain (**3** in comparison with **4** and **8**).

These basic rules were established based on the DPPH, ABTS and similar antioxidant assays and may differ slightly from the results obtained from other tests. Different influence of *O*-methylation on activity is described for FRAP and the inhibition of peroxynitrite induced nitration of tyrosine. The increase of reduction ability in FRAP may be evoked by partial *O*-methylation of the B ring. The presence of an electron-donating moiety on the B ring as the methoxy group confers the higher reducing capability, but total number of hydroxy groups and other mentioned structural properties are necessary and enhance the antioxidant activity of the flavonoids [17].

Previously published studies also show the low effect of *O*-methylation on peroxynitrite induced nitration of tyrosine, when the different position of the *O*-methoxy substitution of antocyanins did not influence their activity [18]. It corresponds with results presented here, which show only a low influence of *O*-methylation on peroxynitrite induced nitration of tyrosine (compare diplacone (**1**) and 3´-*O*-methyldiplacone (**3**)). Results also showed that compounds with *O*-methyl group at similar position on the B-ring (**3**-**8**) have similar antiperoxynitrite activity, while the activity of mimulone (**2**), compound with *p*-OH substituted B-ring is low. Also, the changes on the geranyl side chain did not affect the ability of the compounds tested to prevent tyrosine nitration (**4**, **8**, **10**).

**Table 2:** Antioxidant activities of compounds **1**-**9** determined by using ABTS, DPPH, FRAP, the inhibition of tyrosine nitration (activity expressed as TEAC - ability of the sample to scavenge the radical relative to the radical scavenging ability of Trolox^®^. Value are multiples of activity of Trolox^®^) and superoxide scavenging activity (expressed as % of inhibition at 50 μM concentration).

	ABTS	DPPH	FRAP	Inhibition of. peroxynitrite induced tyrosine nitration	Superoxide scavenging activity	
					Enzymatic	Non-enzymatic
**1**	3.2 ± 0.01	1.06 ± 0.04	0.522 ± 0.01	0.84 ± 0.01	45.2	25.9
**2**	1.7 ± 0.01	0.02 ± 0.01	0.051 ± 0.00	0.09 ± 0.01	-^a^	-^a^
**3**	1.4 ± 0.00	0.12 ± 0.02	0.118 ± 0.00	0.80 ± 0.03	-^a^	-^a^
**4**	1.61 ± 0.01	0.14 ± 0.04	-^a^	0.82 ± 0.01	-^a^	-^a^
**5**	1.62 ± 0.01	0.10 ± 0.00	0.127 ± 0.01	0.74 ± 0.01	-^a^	-^a^
**6**	1.66 ± 0.01	0.98 ± 0.03	0.741 ± 0.01	0.84 ± 0.01	71.2	29.5
**7**	1.60 ± 0.01	0.29 ± 0.02	1.189 ± 0.06	0.83 ± 0.02	-^a^	-^a^
**8**	0.97 ± 0.03	0.12 ± 0.00	-^a^	0.82 ± 0.02	-^a^	-^a^
**9**	-^a^	-^a^	-^a^	0.93 ± 0.02	-^a^	-^a^
**Rutin**	-^a^	-^a^	-^a^	-^a^	50.2	43.6

^a^ Not determined.

### 2.2. Superoxide scavenging activity assay

Both flavonoids **1** and **6** showed a promising scavenging of radicals. According to the scientific literature, there are some requirements in the arrangement of substituents for superoxide scavenging and the inhibition of xanthinoxidase (XO). Searching the literature led however to conflicting conclusions about superoxide scavenging activity and the inhibition of XO by flavanones. Tested samples met the structural conditions for superoxide scavenging: 5,7-dihydroxy substitution, 3´,4´-dihydroxy substitution, and 4-oxo group [19,20]. These conditions are stated for the direct scavenging of superoxide, but not for the inhibition of XO. Some authors state that flavanones are not able to inhibit XO because they lack the *C*-2 - *C*-3 double bond. This double bond causes planarity of the A, C and B rings group due to the conjugation effect and is an important factor for XO inhibition [19]. On the other hand, according to some authors, flavanones might inhibit XO due to the fact that their chemical structure is closely related to hypoxanthine, xanthine, and uric acid, and may act as XO substrate analogs [21].

Based on the results of the ABTS and DPPH assays, the activity of compound **1** and compound **6** was measured using a superoxide-scavenging assay. Two systems for generating superoxide radical were used: enzymatic and non-enzymatic. Both assays, as described in past literature, were primary for the proper evaluation of hydrophilic compound activity because of the aqueous character of the reaction mixture [22]. In this work, the optimization of these assays for hydrophobic compounds (*C*-6 lipophilic substituent) was carried out, and the activity of the compounds tested was compared with rutin at equimolar concentrations of 50 µM.

Some differences between the results of enzymatic and non-enzymatic assays were found (Table 2). In the non-enzymatic test, the activities of the compounds tested were similar (**1** 25.9%, **6** 29.5%), but lower than the activity of rutin (43.6%). The results of the enzymatic assay showed higher activities of **1**, **6** and rutin, in comparison with the non-enzymatic assay. Compound **6** showed the highest activity (71.2%), significantly higher than rutin (50.2%). From these results it can be concluded, that geranylated flavanones can also act as XO inhibitors and, if needed, serve as compounds for the modeling of more potent structures.

### 2.3. Inhibition of Fenton reaction assay

Different methods can be used for the measurement of hydroxyl radical scavenging activity [23]. In this study, the Fenton reaction system generating a hydroxyl radical with plasmid DNA as a detection system was used to bring the assay closer to *in vivo* conditions. Plasmid DNA was used as a target for the attack of OH^•^ radicals. Plasmid DNA is constituted from a circular double strand of nucleic acids in its native supercoiled conformation, also known as CCC (covalently closed circle). The attack of OH^•^ degrades the DNA into series of degradation products. The open circle (OC) conformation is the first step of degradation and it is characterized by having only one strand cut, therefore remaining in its circular conformation. The linear (L) form is the result of a cut in both DNA strands. All these forms are visible by electrophoretic separation.

Oxidative damage of biomacromolecules, including DNA, is considered one of the most dangerous actions of ROS. Plasmid DNA as a model of oxidative disruption was successfully used in several studies [24,25]. The activity of the compounds tested was expressed as a “compounds ratio/ rutin ratio“. Compounds ratio and rutin ratio means ratio of area under curve (AUC), which demonstrates concentration of DNA (see section 3.6), of CCC form /AUC of OC form plus AUC of L form [*i.e.* CCC/(OC+L)]. The higher value means a higher content of CCC-form, thus a lower degradation of plasmid DNA. AUCs of individual plasmid forms were obtained from the densitometric evaluation of electrophoretograms. Among the compounds tested, the highest activity was found for compound **6** (ratio 2,565) and compound **1** (ratio 0,892). The compounds tested can be ordered in decreasing activity: 3´-*O*-methyl-5´-hydroxydiplacone (**6**) > diplacone (**1**) > mimulone (**2**) > 3´-*O*-methyl-diplacone (**3**) > 3´-*O*-methyldiplacol (**5**) > 3´-*O*-methyl-5’-*O*-methyldiplacone (**7**) > 6-isopentenyl-3´-*O*-methyltaxifolin (**10**). Table 3 displays the results of this assay in detail. Among the compounds examined for their antioxidant effect, only the two compounds with the *ortho*-dihydroxy substitution at ring B diplacone (**1**) and 3´-*O*-methyl-5´-hydroxydiplacone (**6**), were significantly active. This may imply that the *ortho* hydroxy substitution is important for the antioxidant activity of such compounds, similarly in the results shown in the DPPH test and FRAP (with exception of compound **7**). 6-isopentenyl-3´-*O*-methyltaxifolin (**10**) showed virtually no antioxidant activity in the Fenton reaction inhibition assay, being the only flavanone tested that lacks the geranyl group (compare **5** and **10**).

**Table 3:** Results of Fenton reaction inhibition assay. Rutin at the molar excess of 50:1 or 10:1 to DNA base pare was compared with the tested compounds at the same molar excess. AUC – area under curve; CCC – covalent closed circle (superhelical form of plasmid); L – linear form of plasmid; OC – open circle (circular form of plasmid).

Compound	AUC			Ratio CCC/ (OC+L)	Compound ratio/Rutin ratio
	CCC	OC	L		
**1** (50:1)	569	1618	98	0.332	0.892
**1** (10:1)	453	3272	458	0.121	0.135
**3 **(50:1)	708	3271	231	0.202	0.543
**3** (10:1)	229	3170	294	0.066	0.073
**7** (50:1)	514	2854	193	0.169	0.454
**7** (10:1)	564	2173	73	0.251	0.279
**Rutin** (50:1)	1288	3207	254	0.372	1
**Rutin** (10:1)	1942	1955	207	0.898	1
**Natural plasmid**	2790	386	193	4.819	-
**2** (50:1)	439	634	-	0.692	0.553
**2** (10:1)	152	1502	-	0.101	0.199
**6** (50:1)	1289	402	-	3.206	2.565
**6** (10:1)	656	1406	-	0.467	0.921
**10** (50:1)	194	1556	96	0.117	0.094
**10** (10:1)	189	1915	122	0.093	0.183
**Rutin** (50:1)	1055	843	-	1.251	1
**Rutin** (10:1)	806	1591	-	0.507	1
**Natural plasmid**	1507	-	-	-	-
**5** (50:1)	505	1379	-	0.366	0.491
**5** (10:1)	-	1373	-	-	-
**Rutin** (50:1)	1155	1493	57	0.745	1
**Rutin** (10:1)	535	1788	47	0.292	1
**Natural plasmid**	2166	258	-	8.395	-

### 2.4. Cytoprotective effect against alloxan-induced diabetes

**Figure 1:**
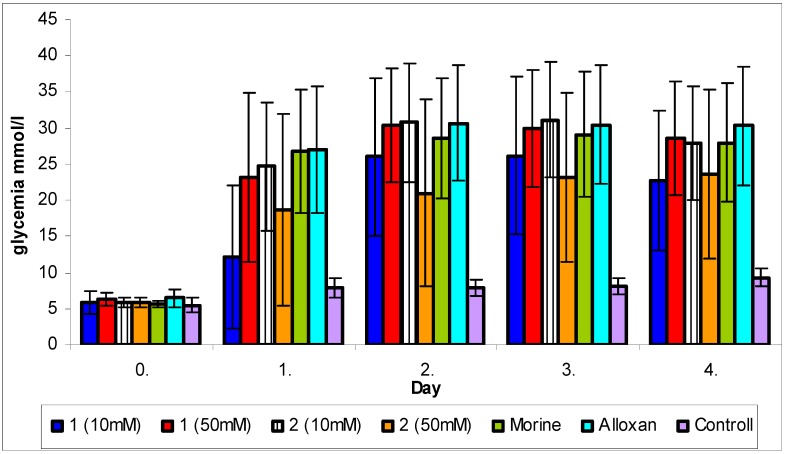
Levels of glycemia mmol/L as determined in alloxan induced diabetes testing of **1** and **2**.

The single *in vivo* test was based on monitoring of cytoprotective effect against alloxan-induced diabetes. Alloxan transported into β-cells of Langerhans islets of mouse pancreas is transformed to dialuric acid, which re-oxidation leads back to alloxan. Several free radicals, such as superoxide, hydroxyl radical or alloxan radical, are formed during this intracellular red/ox process. These free radicals selectively destroy the β-cells, which results in insulin production decrease in treated mice [26]. Flavonoids, as known antioxidants, may prevent the progressive impairment of pancreatic β-cell function due to oxidative stress [27]. Administration of diplacone (**1**) and mimulone (**2**) was not found to reduce the blood glucose levels in alloxan-induced diabetic mice (Figure 1). The only exception was the reduction (p ≤ 0.05) in blood glucose levels of animals treated with diplacone (**1**) on the first day of the experiment. 

Diplacone (**1**) showed significant antioxidant activity in the *in vitro* tests. Whereas the damage of β-cells of Langerhans islets occurs by means of free radicals formed during red/ox reactions (see above), a cytoprotective activity of diplacone (**1**) with consequent decrease of glycemia level was expected in the test focused on cytoprotective effect against alloxan-induced diabetes, as it was described by Soto *et al*. [28]. However, in the present *in vivo* experiment, only minor changes in glycemia level in groups treated with diplacone (**1**) were found. This fact could be caused by low dose of diplacone (**1**) used for experiment [28]. 

**Figure 2:**
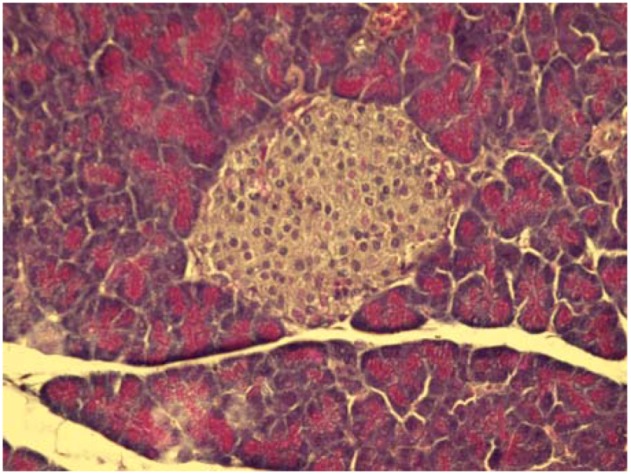
Microphotograph of the pancreas of control mice (HE 400×). Pancreatic islets with distinctly-outlined cell borders (physiological findings).

**Figure 3:**
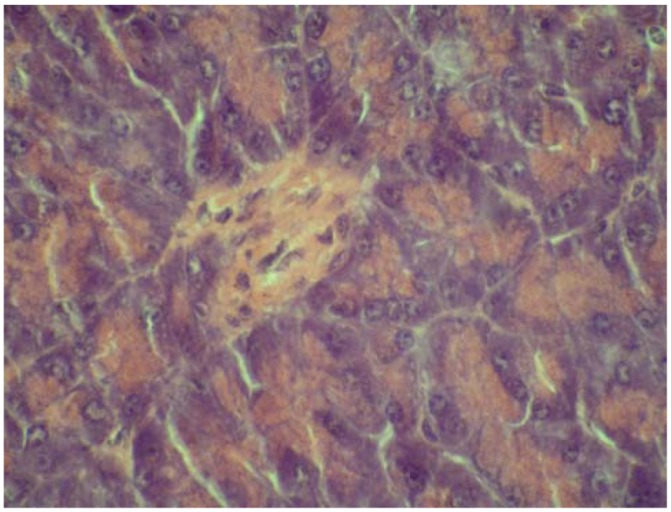
Microphotograph of the pancreas of alloxan-treated mice (HE 600×). Rudimental reaction to the destroyed pancreatic islet.

**Figure 4:**
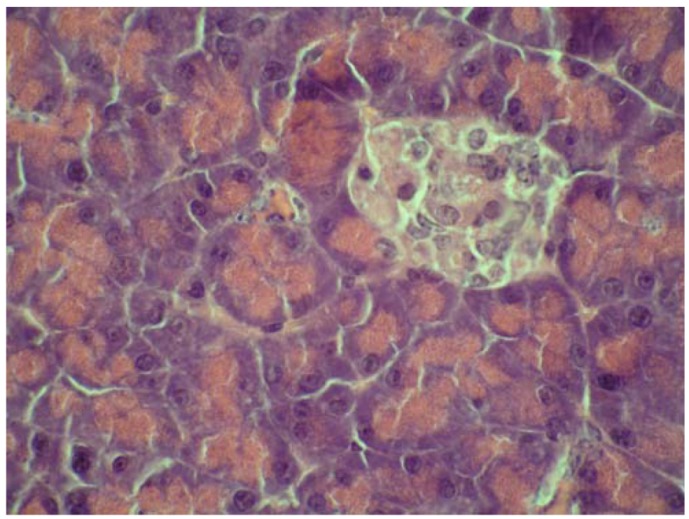
Microphotograph of the pancreas – the effect of mimulone (**2**) on the pancreatic histopathology of diabetic mice (HE 600×). Regressive changes to pancreatic islet cells.

**Figure 5:**
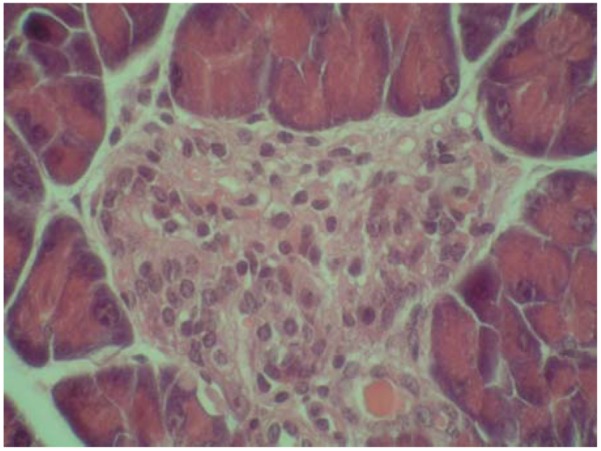
Microphotograph of pancreas – the effect of diplacone (**1**) on the pancreatic histopathology of diabetic mice (HE 600×). Pancreatic islet with an irregular shape; with vacuolised cytoplasm, a pyknotic nucleus and tiny necrosis without a significant inflammatory reaction.

On the other hand, some cytoprotective effect of diplacone (**1**) was proved by histopathological analysis of pancreatic tissue (Figure 2, Figure 3, Figure 4, Figure 5). This observation supports fact that flavonoids may act as a cytoprotective substances [29]. Results of cytoprotective activity assay could be correlated with a higher activity of diplacone (**1**) compared with the other tested compounds.

## 3. Experimental

### 3.1. Tested compounds

*C*-6 geranylated flavonoids **1**-**8** were isolated from *P. tomentosa* fruit [9,10]. Schizolaenone C (**9**) and 6-isopentenyl-3´-*O*-methyltaxifolin **(10)** were isolated from *P. tomentosa* fruit as well (Table 1) [9,30]. As a standard for comparing the activities of the compounds that rutin (Fluka^®^), morine (Sigma-Aldrich^®^) and Trolox^®^ (Sigma-Aldrich^®^) were used. The purity of all the compounds tested was checked *via* HPLC analysis and exceeded 95%.

### 3.2. ABTS and DPPH scavenging activity

The antiradical activity of the compounds was determined spectrophotometrically in the Microplate Reader Synergy HT (Bio-Tek Instruments, Inc) in 96 wells plates in kinetic mode. The DPPH and ABTS assay followed modified methods of Brand-Williams *et al.* [31] and Arnao *et al*. [32], respectively. DPPH test: reaction mixture contained DPPH (63.4 µM) and different concentrations of samples in final volume of 300 µL. Detection at 517 nm. All components were dissolved in methanol. ABTS test: the reaction mixture contained ABTS (83.4 µM, in phosphate buffer pH 7.4) and different concentrations of samples in final volume of 350 µL. Detection wavelength was 734 nm. Samples were dissolved in ethanol. All experiments were performed in triplicate. Activity of the tested compounds was expressed as TEAC.

### 3.3.FRAP

A colorimetric assay, according to Benzie and Strain, was used [33]. Activity was measured spectrophometrically at 593 nm. The reaction mixture contained TPTZ (0.83 mM), FeCl_3_ (1.67 mM) and an acetate buffer (250 mM, pH 3.6). The total volume of the reaction mixture was 3 mL. Samples were measured in triplicates for 6 min in kinetic mode. The results are presented as the equivalent of the Trolox^®^.

### 3.4. Inhibition of peroxynitrite induced tyrosine nitration

A peroxynitrite solution was prepared and an assay carried out according to the method mentioned previously [34]. The percentage of the inhibition of tyrosine nitration was compared to that of the calibrated Trolox^®^ standard. The results are expressed in terms of Trolox^®^ equivalent antioxidant capacity.

### 3.5. Superoxide scavenging activity

Modified enzymatic and non-enzymatic assays described by Valentaõ et al. for the generation of superoxide radical were used [22]. Both methods used spectrophotometric detection at 562 nm, using 96 wells plates and the microplate reader Synergy HT. The assay was performed at room temperature. Samples and standard were measured in equimolar concentrations of 50 µM and subsequently compared. All experiments were performed in triplicate. Activity of the samples was compared with rutin.

#### 3.5.1. Enzymatic assay

The reaction mixture consisted of xanthine (380 µM, in 1 µM NaOH), xanthinoxidase (0.025 U/mL, in 0.1mM EDTA), NBT (42.3 µM, in a 50mM phosphate buffer with 0.1mM EDTA, pH 7.8) and the tested compounds (in DMSO). The final volume of the reaction mixture was 350 µL. The reaction was initiated by the addition of XO and proceeded for 2 min. at room temperature.

#### 3.5.2. Non-enzymatic assay

Superoxide was generated in system NADH/PMS. The reaction mixture contained NADH (166 µM), NBT (43 µM), PMS (2.7 µM), the tested compounds in final volume 300 µL. All components were dissolved in 19 mM phosphate buffer (pH 7.4). Compounds were dissolved in DMSO. The reaction was initiated by the addition of PMS and proceeded for 2 min at room temperature.

### 3.6. Inhibition Fenton reaction assay

This assay was proposed in order to evaluate the capacity of compounds to scavenge OH^•^ and, consequently, to protect DNA from oxidative degradation. To supply OH^•^, a mixture of Fe(III), H_2_O_2_ and ascorbic acid was used. Ascorbic acid reduces Fe(III) to Fe(II), providing a constant flux of OH^•^. The following equations illustrate the Fenton reaction:

Fe^3+^ + ascorbate → Fe^2+^ + oxidized ascorbate

Fe^2+^ + H_2_O_2_ → Fe^3+^ + OH^•^ + OH^-^

OH^•^ + DNA → DNA damage product + OH^-^

**Figure 6:**
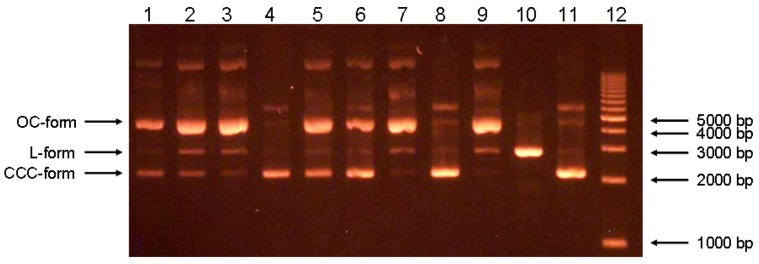
Typical electrophoreogram showing the ability of the individual compound to protect DNA *in vitro*. Lane 1-3: pUC19 + Fenton reaction + **1** (50:1, 10:1, 1:1); Lane 4: pUC19 + **1** 50:1; Lane 5-7: pUC19 + Fenton reaction + rutin (50:1, 10:1, 1:1); Lane 8: pUC19 + rutin 50:1; Lane 9: pUC19 + Fenton reaction + DMSO (vehicle); Lane 10: pUC19 L-form; Lane 11: pUC19 native form; Lane 12: ladder.

The degradation of plasmid DNA was used as a marker of oxidative damage. Supercoiled plasmid pUC19 was isolated from *Escherichia coli* TOP10F´ using a QIAprep Spin Miniprep Kit (QIAGEN, Hilden, Germany). This plasmid (300 ng per reaction) was mixed with compounds dissolved in DMSO and tested in two final ratios of 50:1 and 10:1 (the number of molecules of the tested compound: 1 bp [base pair]), both in the presence or absence of every single intervening agent in the Fenton reaction. Ratio 1:1 was also used, but it was removed from the evaluation because of ambiguous results, which were observed with some compounds. DMSO and rutin were used as negative and positive controls, respectively. A solution of the tested compound was added into a microtube and a TE buffer was used to fill up to the final volume level of 20 µL. This was followed by the addition of 12 µL of the established Fenton reaction mixture, with the final concentrations of 0.66 mM H_2_O_2_, 0.66 mM FeSO_4_ and 0.83 mM ascorbic acid. Reaction mixtures were incubated at 37 ºC for 1 hour and then analyzed using electrophoresis [0.8% agarose gel, voltage 5 V/cm, visualized by ethidium bromide staining (0.15 mg/mL)] (Figure 6). Visualization was performed on a UV transilluminator (λ 312 nm). The electrophoreogram was captured and analyzed by AlphaEaseFC software, version 4.0.0.34 (Alpha Innotech, USA). The relative percentages of circular (CCC) form, one strand nicked (OC) form and linear (L) form of plasmid DNA were evaluated by measurement of the intensity of individual bands. Afterwards, the quantity of different plasmid forms was expressed as an AUC of peaks obtained on the basis of bands intensity. Any compound tested did not digest plasmid, when it was incubated alone with plasmid (data not shown).

### 3.7. Cytoprotective effect against alloxan-induced diabetes

The *in vivo* test of antioxidant activity studied the protective effect of flavonoid compounds from *P. tomentosa* (**1-8**) in mouse model of alloxan-induced diabetes mellitus. This experiment was divided into two steps. First, as a pre-screening, the cytoprotective activity of compounds **1-8** was evaluated (data not shown). According to the pre-screening results we decided to target on the activity of diplacone (**1**) and mimulone (**2**), both at two concentrations (0.5 mmol/kg and 1 mmol/kg body weight).

Female ICR (imprinting control region) albino mice (30–40 g; Anlab, Czech Republic) were used in the experiment. The animals were placed individually in glass metabolic cages at a temperature of 20-24 ºC, fed a standard diet and given water *ad libitum*. The mice were divided into seven groups, each with 10 members: 4 pretreated with the tested compounds in two concentrations, one positive control (only alloxan solution administered), one negative control (isotonic saline solution administered) and one group pretreated with morine known to be a good antioxidant [35].

The tested compounds and alloxan monohydrate were dissolved in a 10% (v/v) DMSO diluted by an *aqua pro injectione*. The solution of the tested compounds was administered to the mice intraperitoneally (at doses of 0.5 mmol/kg and 1 mmol/kg body weight). The alloxan solution (12 mg/mL) was injected into the tail vein (0.1 mL/10 g body weight) 30 min after the application of the tested compounds solution. The experiment was carried out for 5 days, the first day was expressed as day 0. The initial glucose levels were measured in the intact mice. During the next 4 days of the experiment, the glucose levels were measured in the morning after at least 3 h of fasting (Day 2–5). One-drop glucose oxidase test and blood glucose reflective photometer Glucotrend^®^ 2 with Glucotrend^®^ Glucose (max. concentration 33.3 mM; Roche, Germany) test strips were used to determine glucose concentrations (mM) in the venous blood. On the 4th day of the experiment, the animals were destroyed and exsanguinated with pancreas samples taken for histopathological analysis. Samples were fixed in a neutral 10% formol and routinely stained by hematoxyline-eosine. The preparations were examined in an optical microscope, and the observed changes in glucose levels were statistically evaluated using the ANOVA method.

All aspects of animal care complied with the ethical guidelines and technical requirements, and were proven to be consistent with the Animal Scientific Procedures Act 86/609/EC. The state of health of all animals was regularly examined several times a day during both the period of the animal’s acclimation and the whole course of the experiment, by the working team whose members are holders of the Certificate on Professional Competence issued by the Central Commission for the Animal Protection pursuant to § 17 of the Act on Protection of Animals against Cruelty (No. 246/1992 Coll.) of the Czech National Council. 

## 4. Conclusions

The antioxidant activities of different flavonoids with a geranyl (compounds **1-9**) or a prenyl substitution (compound **10**) at position 6 and different substitutions at B and C ring were analyzed. Activity was determined using *in vitro* methods - ABTS, DPPH, FRAP, the inhibition of peroxynitrite-induced tyrosine nitration, superoxide scavenging and the inhibition of Fenton reaction assay. A single *in vivo* test was used for the monitoring of cytoprotective effects of compounds **1** and **2** against alloxan-induced diabetes.

Some of the tested compounds showed notable anti-oxidant activity. The compounds were compared with standards Trolox^®^, rutin, and with morine in the case of the evaluation of cytoprotective effect *in vivo*. Generally, the highest activity was shown to be compounds **1** and **6** with an *ortho* dihydroxy substitution at the B ring of the flavonoid skeleton. The structure vs. antioxidant activity of the evaluated flavonoids was discussed. Studies on the structure-activity relationship have shown and confirmed that the presence of hydroxyl groups and methoxy groups at the A and B rings appear to be important in the antioxidant and free radical scavenging activities of flavonoid compounds. The conclusions of this study have demonstrated that the fruits of *P. tomentosa* provide an efficacious source of natural antioxidant-active substances.
